# Dentofacial Orthopedic Treatment vs. Adenotonsillectomy in Children with Mild to Moderate OSA and Mandibular Retrognathia: A Randomized Controlled Trial

**DOI:** 10.3390/dj14080511

**Published:** 2026-08-11

**Authors:** Yuanyuan Li, Peipei Wang, Anqi Liu, Chen Zhang, Limin Zhao, Liming Yu, Gang Yang, Wei Zhang, Bingjiao Zhao, Xiaoyan Li, Yuehua Liu

**Affiliations:** 1 Sleep Disordered Breathing Center & Department of Pediatric Dentistry, Shanghai Stomatological Hospital & School of Stomatology, Fudan University, Shanghai 200001, China; li_yuanyuan0650@fudan.edu.cn; 2 Shanghai Key Laboratory of Craniomaxillofacial Development and Diseases, Fudan University, Shanghai 200001, China; 3 Department of Orthodontics, Shanghai Stomatological Hospital & School of Stomatology, Fudan University, Shanghai 200001, China; 4 Department of Orthodontics, Shanghai Ninth People’s Hospital, College of Stomatology, School of Medicine, National Clinical Research Center for Oral Diseases, Shanghai Key Laboratory of Stomatology & Shanghai Research Institute of Stomatology, Shanghai Jiao Tong University, Shanghai 200011, China; 5 Department of Otolaryngology and Head and Neck Surgery, Shanghai Children’s Hospital, School of Medicine, Shanghai Jiao Tong University, Shanghai 200001, China; 6 Biomedical Informatics & Statistics Center, School of Public Health, Fudan University, Shanghai 200001, China

**Keywords:** obstructive sleep apnea, dentofacial orthopedic treatment, adenotonsillectomy, upper airway

## Abstract

**Background/Objectives:** Pediatric obstructive sleep apnea (OSA), particularly when accompanied by mandibular retrognathia, presents as a complex, multifactorial condition. Dentofacial orthopedic treatment (DOT) has gained increasing clinical attention; however, high-level evidence specifically demonstrating its efficacy in pediatric OSA remains scarce. This study aimed to evaluate the efficacy of DOT and AT in managing mild to moderate OSA in children with mandibular retrognathia. **Methods:** This open-label, randomized controlled clinical trial recruited 93 children aged 7–10 years with mild to moderate OSA (an AHI of 1–10 events per hour) and mandibular retrognathia (ANB angle ≥ 4.5°). Participants were randomly allocated to four groups: pharmacotherapy, DOT (combined maxillary expansion and mandibular advancement appliance), AT (adenotonsillectomy under general anesthesia), and AT & DOT groups. The primary outcome was the change in apnea–hypopnea index (AHI) from baseline to 7 months post-treatment. Secondary outcomes included assessments of dentofacial development and volumetric changes in the upper airway. **Results:** Because of the high dropout/loss-to-follow-up rates in the pharmacotherapy and AT plus DOT groups, the final analysis was restricted to the DOT and AT groups, in which the overall dropout/loss-to-follow-up rate was 19.6%. Among the 43 participants allocated to the DOT (8 girls and 15 boys) and AT groups (8 girls and 12 boys), 36 completed the follow-up, the median baseline AHI was 3.7 events/h [IQR, 1.9–4.4]. Per protocol analysis revealed a mean reduction in AHI of 1.6 events/h in the DOT group and 1.3 events/h in the AT group, with no significant difference between the groups (−0.43 [95% CI, −1.48 to 0.61], *p* = 0.40). DOT significantly promoted sagittal mandibular growth without inducing a vertical clockwise rotation tendency, whereas AT had no significant effect on dentofacial development. Total upper airway volume increased significantly in both groups, with the increase concentrated in different regions of the upper airway depending on the group. **Conclusions:** In children with mild to moderate OSA and mandibular retrognathia, DOT demonstrated comparable efficacy to AT, which may be attributable to favorable anatomical remodeling of the upper airway.

## 1. Introduction

Obstructive sleep apnea (OSA) in children refers to recurrent episodes of partial or complete obstruction of the upper airway during sleep, resulting in a range of pathophysiological alterations that disrupt normal ventilation and sleep patterns. A 2010 report from Hong Kong revealed that the prevalence of OSA was 5.8% in boys and 3.8% in girls [1]. Over the last decade, the incidence of pediatric OSA has been on the rise, increasing from below 10% to a range of 10–20% [2].

Pediatric OSA is linked to a variety of possible complications, such as dentofacial abnormalities, attention deficit/hyperactivity disorder, learning difficulties, and growth problems [3]. The repercussions of untreated OSA in children extend beyond disruptions to physiological and neuropsychological well-being, potentially causing long-term negative effects on health that may persist into adulthood.

The causes of pediatric OSA are diverse, with adenotonsillar hypertrophy being the most common risk factor [4]. Other factors contributing to OSA include dentofacial deformities and obesity, both of which can exacerbate the narrowing and collapse of the upper airway [5]. A range of treatment options is available to reduce the apnea–hypopnea index (AHI), such as adenotonsillectomy (AT) [6,7,8], mandibular advancement (MA) [9], rapid maxillary expansion (RME) [10], pharmacological treatments [11], myofunctional therapy [12], anti-inflammatory drugs [13], weight management plans, and continuous positive airway pressure (CPAP). However, not all treatments provide satisfactory results or are well tolerated by patients. Among these, AT is a widely recognized first-line treatment for pediatric OSA in multiple guidelines [14]. Despite AT’s effectiveness, its results can vary among children with OSA [6]. Research has suggested that pediatric OSA is frequently not fully resolved by AT alone [15]. Although many children experience significant improvements in PSG parameters after surgery, a substantial proportion still do not achieve complete normalization [16]. In the PATS trial, 2.2% of children who underwent AT experienced severe complications related to the surgery [8]. Patients with mild OSA or high surgical risk may require non-surgical interventions such as orthodontic treatment or respiratory support [17].

Dentofacial deformities, such as retrognathia, are frequent complications of pediatric OSA and are often linked to a narrow maxillary width. These dentofacial features can either contribute to the development of OSA or result as a secondary effect of prolonged abnormal oral breathing due to OSA. RME and MA both belong to dentofacial orthopedic treatment (DOT), capable of correcting maxillary constriction and mandibular retrognathia, as well as expanding the upper airway to manage pediatric OSA [17]. The American Thoracic Society document Management of Persistent, Post-adenotonsillectomy Obstructive Sleep Apnea in Children: An Official American Thoracic Society Clinical Practice Guideline stated that maxillary and mandibular advancement are commonly used to treat pediatric OSA [18]. It also recommends orthodontic treatment and DOT for children with persistent OSA and specific dentofacial features. The updated AAO white paper, published in AJODO, emphasizes that orthodontists must consider potential skeletal differences when making treatment decisions for patients with SDB [19]. However, to date, there remains a lack of high-level evidence in evidence-based medicine demonstrating the effectiveness of this approach in OSA children with mandibular retrognathia.

This trial aimed to evaluate the effectiveness of DOT (MA combined with RME) compared to AT in children with mild to moderate OSA and mandibular retrognathia, providing a comprehensive perspective and supporting evidence for clinical decision-making. This trial, to the best of our knowledge, is the first randomized controlled trial to directly compare the effects of DOT versus AT in OSA children.

## 2. Materials and Methods

Design and participants:

This study is an open-label, non-blinded, randomized trial taken place between March 2018 and December 2020 at two tertiary hospitals in Shanghai, China: Shanghai Stomatological Hospital and Shanghai Children’s Hospital. Reporting of this RCT followed the CONSORT 2025 guidelines, with the full checklist available in Table S1.

Eligible children were between the ages of 7 and 10 years, diagnosed with mild to moderate OSA, defined by an AHI of 1–10 events/h, and met criteria for mandibular retrognathia (Angle ANB ≥ 4.5°) and adenotonsillar hypertrophy (diagnosed by both orthodontists and ENT specialists). Exclusion criteria included central sleep apnea (CSA), nasal stenosis, and a body mass index (BMI) z-score of 3 or higher, calculated as weight divided by height squared.

Written informed consent was obtained from all participants. Children were randomly assigned in a 1:1:1:1 ratio to receive pharmacotherapy, DOT, AT surgery, or AT followed by postoperative DOT (AT & DOT group). Patients in the AT group received endoscopy-assisted AT under general anesthesia. Patients in the DOT group were treated using a removable Twin-block appliance for mandibular advancement combined with RME, according to the standardized protocol described in the published study protocol [20]. Briefly, the appliance was inserted and worn for a 1-month adaptation period without activation of the expansion screw. Thereafter, the screw was activated twice daily until transverse overcorrection was achieved. Patients were instructed to wear the appliance full-time, except during meals and tooth brushing. The clinical endpoint of treatment was defined as sagittal correction toward a near-Class I molar relationship or an edge-to-edge incisal relationship, with concurrent transverse arch coordination in the advanced mandibular position. The appliance was maintained until the clinician determined that the active treatment phase had been completed.

Study Oversight:

Recruitment announcements were made at the participating hospitals and on their official websites. Written informed consent was obtained from the guardians of participants by trained clinical research assistants, who provided a detailed explanation of the study. Randomization was performed using a central system developed by KNOWLANDS, applying the minimization method with an 80% allocation probability. The randomization was based on stratification factors including sex and obesity status (defined by BMI z-score), and was carried out using researchers’ mobile devices. Monitoring groups from the Shanghai Shenkang Hospital Development Center conducted semi-annual site visits to ensure compliance, verifying that case report forms (CRFs) were completed correctly and in line with the original records.

Outcomes:

PSG assessments were carried out at sleep monitoring centers at baseline (M0) and 7 months after treatment (M7). Additionally, X-ray lateral cephalometric radiographs and cone-beam computed tomography (CBCT) scans were performed at the Radiology Department of Shanghai Stomatological Hospital. The digitized X-ray and CBCT images were analyzed using Dolphin Imaging software (Version 11.95, Dolphin Imaging & Management Solutions, Chatsworth, CA, USA), with evaluations conducted by researchers who were blinded to the treatment groups.

**Figure 1:**
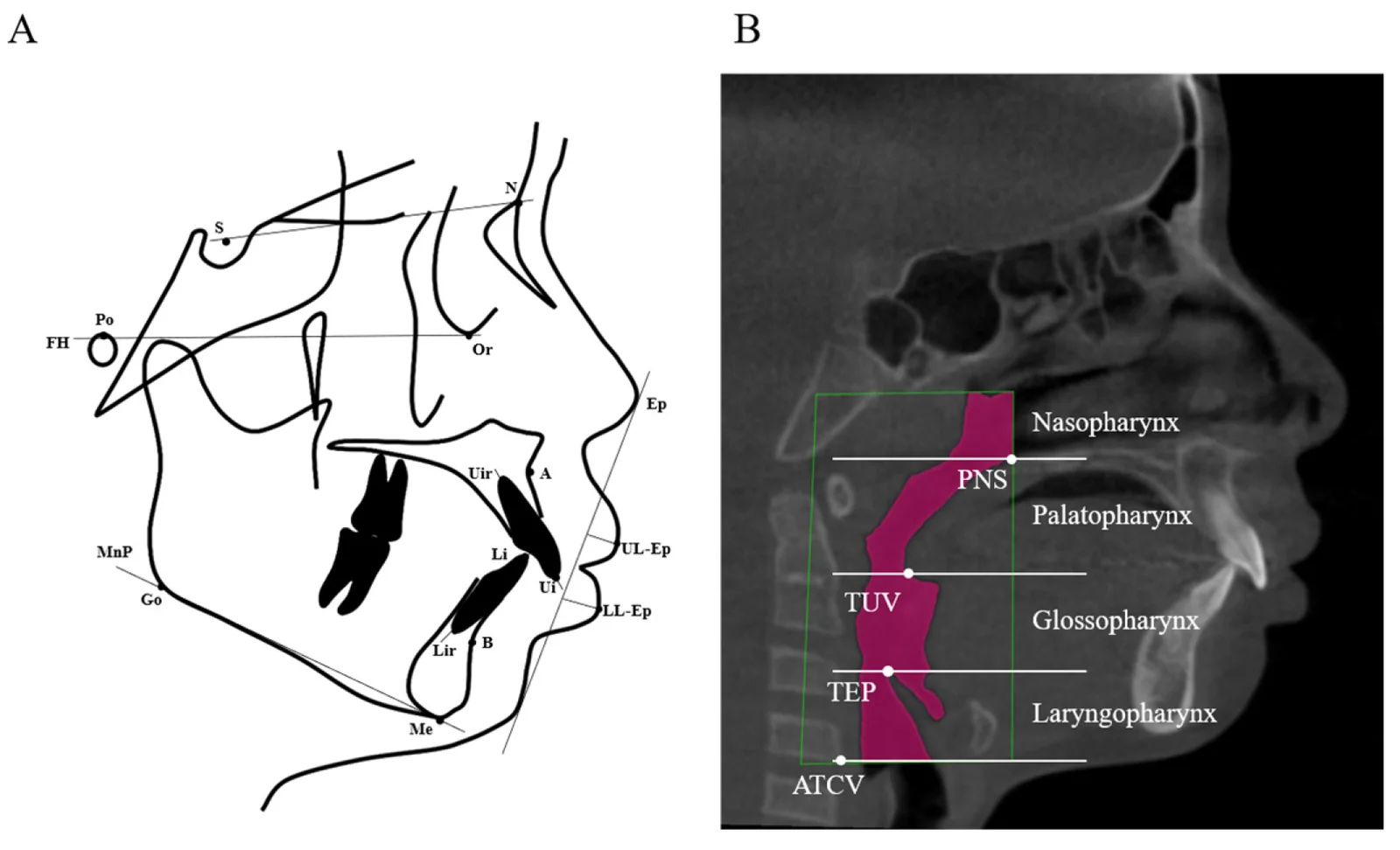
Cephalometric landmarks and indicators from X-ray and morphologic analysis of the upper airway from CBCT. (**A**) Cephalometric landmarks and indicators. S, sella; N, nasion; Po, porion; Or, orbitale; A, subspinale; B, supramental; Ui, upper incisor; Uir, upper incisor root; Li, lower incisor; Lir, lower incisor root; Me, menton; Go, gonion; UL, upper lip anterior; LL, lower lip anterior; FH, Frankfort plane; MnP, the mandibular plane; Ep, the aesthetic plane; UL-Ep, distance between UL and the aesthetic plane; LL-Ep, distance between LL and the aesthetic plane. (**B**) Morphologic analysis of upper airway. PNS, posterior nasal spine; TUV, tip of the uvula; TEP, tip of the epiglottis; ATCV, anteroinferior aspect of the vertebral body of the fourth cervical vertebra; AP, anteroposterior.

The primary outcome was the change in the AHI from M0 to M7. AHI was defined as the average number of apneas and hypopneas per hour during sleep. Mixed sleep apnea events were included in the data, but central sleep events were excluded. Secondary outcomes included changes in LSaO_2_, dentofacial development assessed through X-ray cephalometric measurements (including 7 angular measurements and 2 linear measurements), and volumetric changes in the upper airway segments based on CBCT reconstructions. The cephalometric measurements from X-ray and upper airway measurements from CBCT are presented in Figure 1. Detailed descriptions of the measurement methods and parameters are provided in prior reports [20].

Sample Size:

The sample size calculation assumed a conservative 20% attrition rate for the four groups. We hypothesized that the intervention may decrease subjects’ AHI. With reference to previous studies, including a nonrandomized study, a sample of 30 cases per group allowed us to detect an effect size of 0.8 (AHI decreases of 2.0, 3.7, 3.7, and 4.0 after therapy, respectively, with a standard deviation 2.95) [21].

Statistical Analysis:

Data analysis was performed using the Full Analysis Set (FAS) and Per-Protocol Set (PPS). The primary outcomes were presented as treatment changes within the FAS and PPS. Secondary outcomes were analyzed using the FAS when data were available; participants without M7 CBCT data were excluded from CBCT-based airway analyses. For participants lost to follow-up, missing data were addressed by using baseline carryover methods for imputation, and these imputed data were included in the secondary outcome analyses. Statistical analysis was conducted using IBM SPSS Statistics for Windows, version 27.0 (IBM Corp., Armonk, NY, USA). Continuous and categorical variables were summarized as means (SD) or frequencies (percentages) as appropriate. Changes from M0 to M7 in each group were evaluated using paired-sample *t*-tests, while inter-group comparisons were made using analysis of covariance, adjusting for the corresponding baseline value. All statistical tests were two-sided, with a *p* value of <0.05 considered statistically significant.

## 3. Results

### 3.1. Study Overview

**Figure 2:**
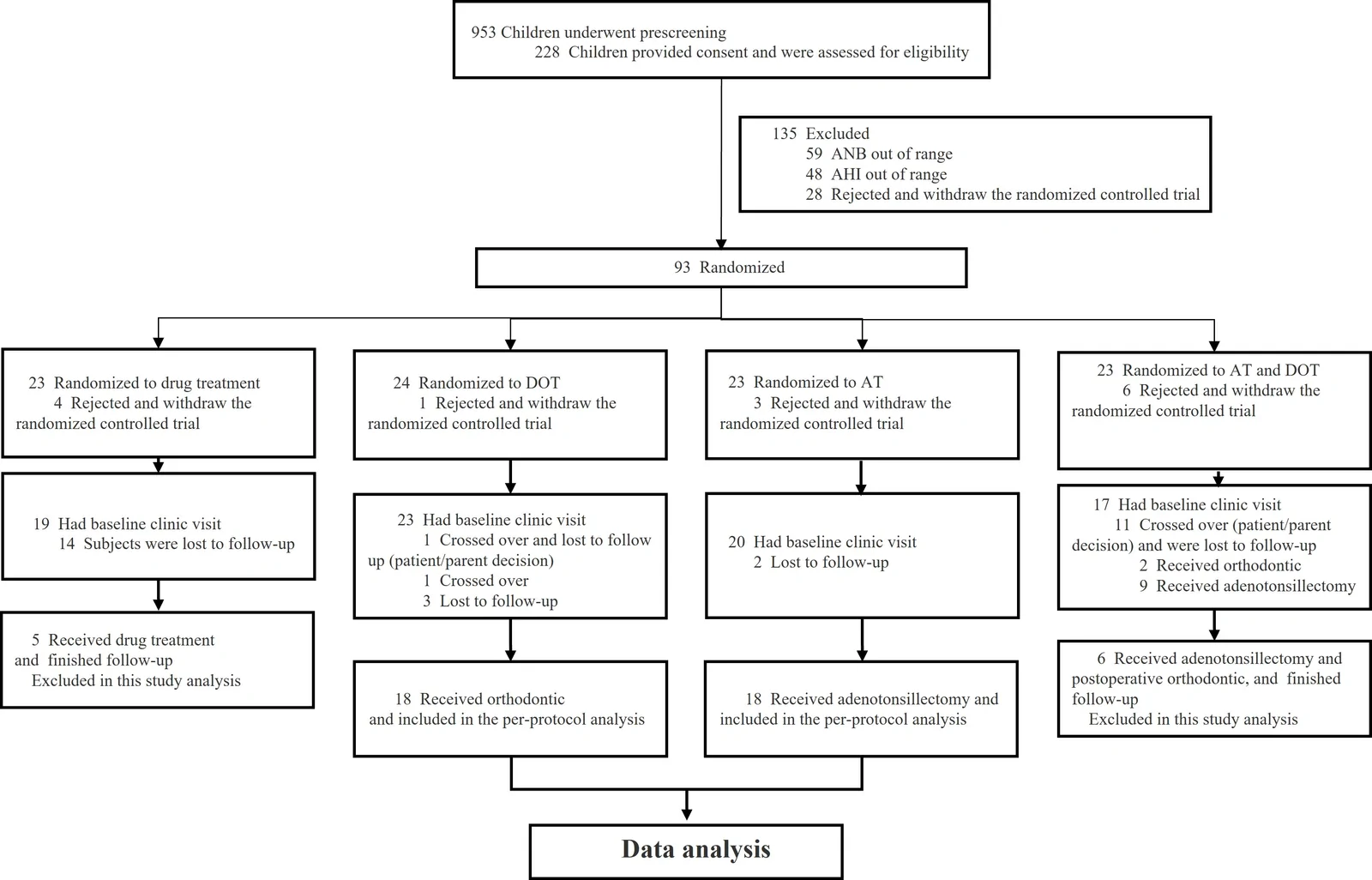
Study Flow Diagram.

Figure 2 illustrates the participant flow in the study. From March 2018 to December 2020, the research team initially screened 953 children. A total of 227 children and their guardians consented to participate and underwent further evaluations; 93 participants were ultimately enrolled. Recruitment and follow-up were significantly impacted by the global COVID-19 pandemic. The dropout/loss-to-follow-up rates in the pharmacological group and AT plus DOT groups reached 78.3% and 73.9%, respectively. In the DOT group, 19 subjects (79.2%) finished the treatment, and primary endpoint data were gathered, with one subject violating the randomization protocol. In the AT group, 18 subjects (81.8%) completed the treatment and provided data for the primary outcome. As a result, only the DOT group and the AT group were included in the final analysis, leading to an overall dropout/loss to follow-up rate of about 19.6%.

### 3.2. Baseline Characteristics

The baseline characteristics of the study groups are presented in Table 1. Of the participants, 16 were female (37.2%) and 27 were male. The average age for children in the DOT group was 8.2 ± 1.1 years, and in the AT group, it was 7.5 ± 1.3 years. Among the FAS cohort, 31 participants (72.1%) reported a mouth-breathing habit, while 35 children (81.4%) exhibited snoring. Of the participants, 36 had tonsil hypertrophy classified as Grade II, and 7 had Grade III hypertrophy. Additionally, 31 children had moderate adenoid hypertrophy (A/N > 0.6), with 60–80% hypertrophy, and 12 subjects had adenoid hypertrophy exceeding 80%. There was no statistically significant difference in baseline AHI between the two groups, and the demographic and clinical characteristics at baseline were well-matched.

### 3.3. Primary Outcome

The comparative results between the two study groups are summarized in Table 2. In the FAS cohort, the average AHI score in the DOT group decreased by 1.23 events/h (95% CI, −1.84 to −0.62) from M0 to M7, while the AT group showed a reduction of 1.19 events/h (95% CI, −2.27 to −0.10). The difference between the groups was −0.06 events/h (95% CI, −1.01 to 0.89), which was not statistically significant (*p* = 0.90).

In the PPS analysis, the average AHI score in the DOT group dropped by 1.57 events/h (95% CI, −2.27 to −0.87), while the AT group had a reduction of 1.32 events/h (95% CI, −2.52 to −0.12). No significant differences were found between the two groups (−0.43 events/h [95% CI, −1.48 to 0.61], *p* = 0.40).

**Figure 3:**
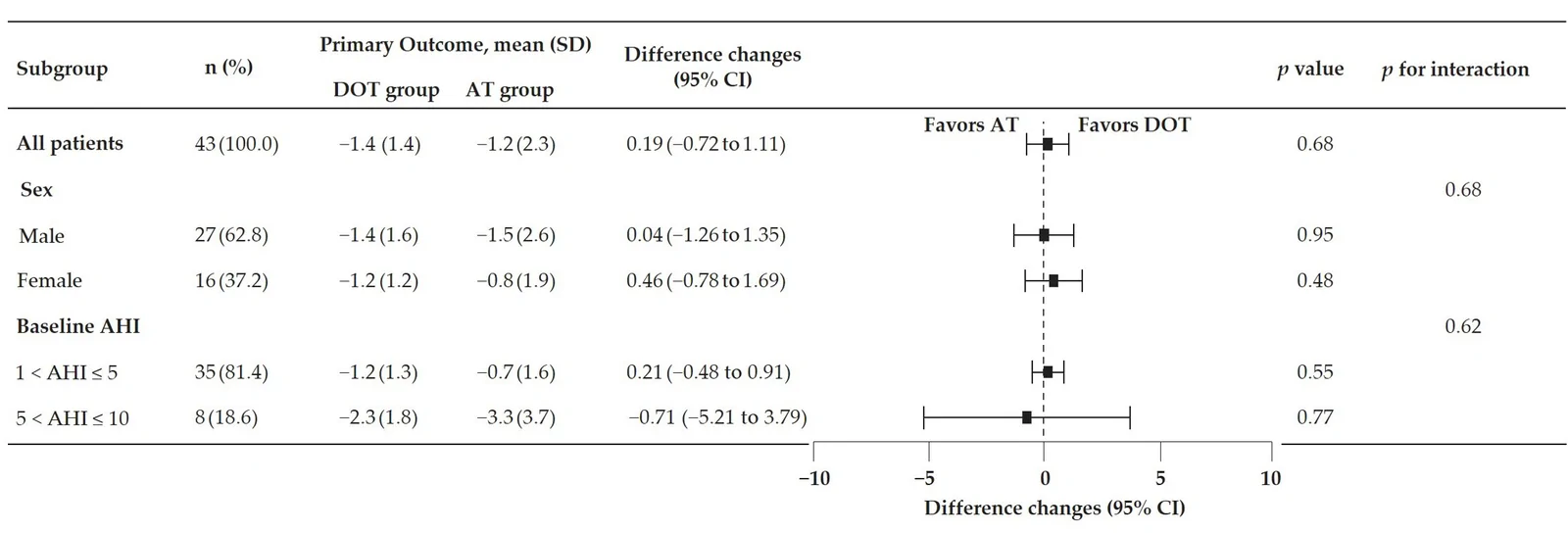
Subgroup analysis of primary outcome in the FAS. Abbreviations: CI: Confidence Interval; AHI: apnea–hypopnea index, reported in events/h.

Further, exploratory subgroup analyses were performed based on gender and the severity of OSA in the participants to assess the effects of DOT and AT on the primary outcome across different subgroups (Figure 3). The results showed that the efficacy of both treatments was not affected by the characteristics of gender and severity of OSA at M0 (*p* > 0.05).

### 3.4. Secondary Outcomes

The secondary outcomes for both groups were analyzed using the FAS dataset (Table 3). A total of 36 participants provided PSG data and lateral cephalometric radiographs at both M0 and M7. However, 34.9% of participants declined to undergo CBCT scans at M7, which led to their exclusion from the analysis of changes in the upper airway. In the DOT group, LSaO_2_ showed a modest improvement of 5.00% (95% CI, 0.07% to 10.07%) from M0 to M7. The AT group experienced an increase of 3.72% (95% CI, 0.52% to 6.93%). The difference between the groups was 1.21% (95% CI, −1.78% to 4.21%), which was not statistically significant (*p* = 0.42).

Among the participants receiving DOT, the SNB angle, a measure of forward sagittal growth of the mandible, significantly increased by 1.04° (95% CI, 0.42° to 1.65°). In contrast, the AT group showed a minor change of 0.17° (95% CI, −1.03° to 1.36°). The change in the ANB angle was more pronounced in the DOT group (−1.53° [95% CI, −2.09° to −0.79°]) compared to the AT group (−0.12° [95% CI, −0.86° to 0.63°]), with a difference between the groups of −2.94° (95% CI, −5.45° to −0.43°) (*p* = 0.023). Regarding vertical growth indicators of the mandible, namely SN-MP and FH-MP, no significant differences were observed between the two groups. Additionally, analyses of the incisors inclination and lip protrusion showed no significant differences either within or between the groups before and after treatment. These results suggest that DOT effectively promoted forward sagittal growth of the mandible without inducing a tendency for vertical clockwise rotation.

In Table 3, both groups show a significant increase in total upper airway volume, with the DOT group experiencing an increase of 2696.8 mm^3^ (95% CI, 809.09 mm^3^ to 4584.57 mm^3^) and the AT group demonstrating a larger increase of 3743.9 mm^3^ (95% CI, 1745.84 mm^3^ to 5742.04 mm^3^). However, no significant differences were found between the two groups. In the DOT group, the nasopharynx and palatal pharynx volumes significantly increased after treatment, along with a notable enlargement in the glossopharynx cross-sectional area. Similarly, the AT group showed significant increases in the cross-sectional areas and volumes of the nasopharynx and palatopharynx, while no substantial changes were observed in the glossopharynx and laryngopharynx. Comparative analysis revealed that the DOT group had smaller increases in total upper airway volume and the volumes of the nasopharynx and palatal pharynx than the AT group, although these differences were not statistically significant. Notably, the DOT group exhibited a greater increase in the cross-sectional area of the oropharynx compared to the AT group, with an inter-group difference of 37.99 mm^2^ (95% CI, 13.53 mm^2^ to 62.45 mm^2^), *p* < 0.01. These findings indicate that while both DOT and AT had similar effects on total upper airway volume, they produced varying degrees of widening in different parts of the upper airway.

### 3.5. Safety

No serious adverse events were reported in this study. Among participants in the AT group, two children experienced mild postoperative bleeding, which was related to the surgical procedure. In the DOT group, one child reported a sensation of a foreign body, while two others developed gingivitis due to poor oral hygiene. These issues resolved after appropriate interventions, including guidance on appliance wear, dental cleaning, and oral hygiene education. Thus, these findings suggest that both AT and DOT were generally safe but highlight the importance of proper guidance on appliance use for children undergoing DOT.

## 4. Discussion

OSA is a complex condition in children, often resulting from multiple factors that contribute to upper airway obstruction, including adenoid hypertrophy, tonsillar enlargement, and structural narrowing due to dentofacial deformities. As such, the diagnosis and treatment of pediatric OSA typically require collaboration from a multidisciplinary team. In cases of severe OSA, children commonly exhibit significant adenotonsillar hypertrophy, making combination therapy, including AT surgery, the preferred treatment option [22,23]. However, the necessity of AT surgery for mild to moderate OSA in children remains a topic of debate. This controversy arises from two main considerations: firstly, adenoids and tonsils often naturally shrink with age, prompting some clinicians to be more cautious about recommending AT under general anesthesia [24]. Secondly, for children with severe underlying conditions such as dentofacial abnormalities, the residual AHI after AT surgery can remain alarmingly high, sometimes reaching 70% [25].

This trial, to the best of our knowledge, is the first randomized controlled trial to directly compare the effects of DOT versus AT in children with mild to moderate OSA, encompassing three critical dimensions: PSG data, dentofacial development, and upper airway structure. The results of this study indicated that, for children with mild to moderate OSA and mandibular retrognathia, there were no significant differences between DOT and AT in terms of improving AHI and LSaO_2_. However, DOT notably stimulated forward sagittal growth of the mandible, which in turn led to an enlargement of the nasopharynx, palatal pharynx, and glossopharynx. In contrast, the AT group showed no significant changes in dentofacial development, but the removal of hypertrophied soft tissue resulted in a significant increase in the volumes of the nasopharynx and palatal pharynx. These findings lay the groundwork for a multidisciplinary approach to pediatric OSA management, offering valuable insights into the distinct contributions of DOT and AT interventions.

Previous research has primarily focused on maxillary expansion or mandibular advancement individually for pediatric OSA, with both treatments demonstrating stable long-term outcomes [10,26,27]. However, children in the transitional dentition stage often present with concurrent maxillary constriction and mandibular retrognathia [28]. In clinical practice, maxillary expansion can be effectively integrated with various mandibular advancement appliances to augment the maxilla’s transverse width. Consequently, the orthopedic strategy employed in this study incorporated the use of a removable Twin-block appliance in tandem with RME. This combination effectively expanded the maxillary arch, thereby increasing the transverse dimensions of the nasopharynx [29], while also advancing the mandible. This dual action leads to enhancements in the sagittal dimensions of the palatal pharynx and glossopharynx [26,30], offering a comprehensive approach to address the complex anatomical challenges associated with pediatric OSA.

In this study, both treatment groups showed notable improvements in sleep -related respiratory function, but residual AHI remained present. It is widely accepted that factors such as obesity [16,31,32,33], preoperative severe OSA [33,34], tonsillar hypertrophy [27], age [16], male gender [31], and serious comorbidities [32] are linked to residual AHI after surgery. A follow-up study spanning 36 months demonstrated that AHI levels increased over time in 68% of cases after AT [35]. However, few previous studies have addressed whether dentofacial deformities, such as mandibular retrognathia, are associated with suboptimal postoperative results following AT.

In this study, the AHI decreased by −1.23 (−1.84 to −0.62) in the DOT group and by −1.19 (−2.27 to −0.10) in the AT group, with no significant differences between the two groups (Table 2). It is important to note that only one patient in the AT group achieved complete resolution of OSA (AHI < 1 events/h) at the M7 follow-up, whereas three patients in the DOT group reached the same AHI threshold. The proportion of participants with residual AHI in both groups was notably higher than what has been reported in previous studies. We suggest that this could be due to the inclusion of patients with both adenotonsillar hypertrophy and mandibular retrognathia, indicating that no single treatment method may be sufficient to address the narrowing of the upper airway. A systematic review has shown that combining AT with DOT is more effective than using either treatment alone, leading to a significant reduction in AHI in pediatric OSA patients [34]. Given the complexity of OSA, additional research is necessary to better comprehend its underlying mechanisms and to refine treatment approaches.

DOT not only alleviates OSA symptoms but also improves the facial appearance of patients with mandibular retrognathia linked to OSA [26]. As anticipated, children who received DOT showed favorable changes in the relative position of the mandible, whereas no significant alterations were noted in the AT group. The changes in the SNB and ANB angles were more significant in the DOT group compared to the AT group, with the difference between groups being 0.93° (95% CI, −0.23° to 2.09°) (*p* = 0.11) and −2.94° (95% CI, −5.45° to −0.43°) (*p* < 0.05), respectively. No meaningful differences were observed between the groups in terms of the mandibular plane angles, MP-SN and MP-FH angles (Table 3). These findings suggested that DOT, as applied in this trial, significantly promoted forward sagittal growth of the mandible without increasing the tendency for vertical clockwise rotation.

Longitudinal studies on nasopharyngeal growth have shown that the rate of growth of nasopharyngeal lymphoid tissue is faster than that of the nasopharynx itself between the ages of three and five, leading to a reduction in airway volume [36]. By the age of seven, the adenoids begin to shrink, resulting in a corresponding increase in airway volume [37]. The impact of DOT on the nasopharynx likely involves both the effects of RME and natural growth processes. In this study, the increase in upper airway volume associated with DOT affected the nasopharynx, palatal pharynx, and glossopharynx, while AT mainly influenced the nasopharynx and palatal pharynx. These anatomical changes in the upper airway may explain why DOT in children with OSA and mandibular retrognathia can lead to PSG improvements.

There is a two-way relationship between OSA and oral health [38]. Orthodontists are increasingly playing a key role in the screening and management of OSA [39]. This study integrates the examination of jaw development, occlusion, and profile morphology into OSA research, providing valuable insights and preliminary findings. However, several limitations should be acknowledged. One of the major drawbacks is the interruption of the research due to social circumstances and the COVID-19 pandemic, which led to a smaller final sample size than initially planned, potentially limiting the reliability of the results. Despite this, the randomized controlled design and the blinding of key analysts provide a useful reference for future research. Additionally, the study initially included a pharmacotherapy group and an AT followed by postoperative DOT group to evaluate the effectiveness of a combined approach for this multifactorial condition. However, due to a high dropout rate in the drug treatment group and high dropout/loss-to-follow-up rate in the AT followed by postoperative DOT group, both of these groups were ultimately excluded from the final efficacy analysis. Furthermore, while the durations of AT surgery and DOT differed, a 7-month post-treatment follow-up was used as the primary endpoint for both groups, which may have influenced the study results. Given the chronic and progressive nature of the disease, future studies should incorporate longer follow-up periods to evaluate the long-term effectiveness of OSA interventions. Based on this research, our team is initiating a non-inferiority randomized controlled trial to assess the effectiveness of AT and DOT in patients with OSA associated with mandibular retrognathia [40].

## 5. Conclusions

In children with mild to moderate OSA and mandibular retrognathia, both DOT and AT led to improvements in the AHI, with no significant differences in efficacy between the two approaches. These improvements may be attributed to anatomical changes in the upper airway. Additionally, DOT and AT have distinct effects on dentofacial development and various sections of the upper airway. However, further studies with larger sample sizes and control groups are needed to confirm these findings.

## Figures and Tables

**Table 1:** Baseline Characteristics of Participants.

Characteristic	Per-Protocol Set Population		Full Analysis Set Population	
	DOT Group (*n* = 18)	AT Group (*n* = 18)	DOT Group (*n* = 23)	AT Group (n = 20)
Sex, No. (%)				
Female	6 (33)	8 (44)	8 (35)	8 (40)
Age, mean(SD), y	8.1 (1.3)	7.6 (1.4)	8.2 (1.1)	7.5 (1.3)
Height, mean (SD), cm	126.7 (11.2)	121.8 (12.0)	128.2 (10.7)	121.4 (11.5)
Weight, mean (SD), kg	28.9 (6.6)	26.0 (7.4)	29.5 (6.1)	25.7 (7.1)
BMI, mean(SD), kg/m^2^	17.8 (1.5)	17.2 (1.3)	17.8 (1.4)	17.1 (1.3)
AHI, mean(SD), events/h	3.9 (2.5)	3.6 (2.1)	3.6 (2.0)	3.7 (2.5)
SNA, mean (SD), °	79.9 (3.8)	80.1 (4.4)	79.7 (3.5)	79.9 (4.3)
SNB, mean (SD), °	73.5 (4.0)	73.5 (3.6)	73.3 (3.8)	73.1 (3.7)
ANB, mean (SD), °	6.4 (1.9)	6.6 (2.5)	6.3 (1.7)	6.8 (2.5)
Rhinitis, No. (%)	18 (100)	18 (100)	23 (100)	20 (100)
Snore, No. (%)	16 (89)	15 (83)	20 (87)	15 (75)
Mouth breathing, No. (%)	17 (94)	13 (72)	17 (74)	14 (70)
Tonsil grade, No. (%) ^a^				
II	17 (94)	15 (83)	20 (87)	16 (80)
III	1 (6)	3 (17)	3 (13)	4 (20)
Adenoidal hypertrophy, No. (%) ^b^				
60–80%	14 (78)	13 (72)	16 (70)	15 (75)
>80%	4 (22)	5 (28)	7 (30)	5 (25)

Abbreviations: BMI: body mass index, calculated as weight in kilograms divided by height in meters squared (kg/m^2^); AHI: apnea–hypopnea index. SNA, °: angular indicator for assessment of maxillary protrusion; SNB, °: angular indicator for assessment of mandibular protrusion; ANB, °: angular indicator for assessment of the sagittal relationship between the jaws. ^a^ Tonsil grade: Grade I hypertrophic tonsils do not exceed the palatoglossal arch and velopharyngeal arch; grade II: beyond the palatopharyngeal arch but not reaching the midline of the posterior pharyngeal wall; Grade III: beyond the midline or bilateral tonsils touching each other. ^b^ Adenoidal hypertrophy: Expressed using the ratio of adenoid thickness to nasopharyngeal cavity (A/N); A: adenoid thickness, the distance from the most convex point of the adenoid to the tangent line of the anterior edge of the occipital slope; N: nasopharyngeal width, the distance from the posterior superior of the hard palate to the intersection of the pterygium and the base of the skull.

**Table 2:** Primary outcome in the FAS and PPS analysis.

Variable	DOT Group			AT Group			Between-Group Difference in Change (95% CI)	*p* Value
	M0	M7	Changes (95% CI)	M0	M7	Changes (95% CI)		
FAS	n = 23	n = 23	n = 23	n = 20	n = 20	n = 20		
AHI, mean (SD), events/h	3.60 (2.0)	2.41 (1.67)	−1.23 (−1.84 to −0.62) **	3.7 (2.5)	2.48 (2.11)	−1.19 (−2.27 to −0.10) *	−0.06 (−1.01 to 0.89)	0.90
PPS	n = 18	n = 18	n = 18	n = 18	n = 18	n = 18		
AHI, mean (SD), events/h	3.56 (2.05)	1.98 (1.35)	−1.57 (−2.27 to −0.87) ***	3.88 (2.52)	2.56 (2.21)	−1.32 (−2.52 to −0.12) *	−0.43 (−1.48 to 0.61)	0.40

Abbreviations: CI: Confidence Interval; FAS: Full Analysis Set; PPS: Per-Protocol Set; AHI: apnea–hypopnea index, reported in events/h. Intra-group changes were assessed using paired *t*-tests; analysis of covariance (ANCOVA) was used for comparisons between groups. * *p* < 0.05; ** *p* < 0.01; *** *p* < 0.001.

**Table 3:** Secondary outcomes in the FAS dataset.

Variable	DOT Group			AT Group			Difference Changes (95% CI)	*p* Value
	M0	M7	Changes (95% CI)	M0	M7	Changes (95% CI)		
PSG data								
LSaO_2_, mean (SD), %	84.7 (7.4)	89.7 (4.4)	5.00 (0.07 to 10.07)	84.8 (5.2)	88.5 (4.5)	3.72 (0.52 to 6.93) *	1.21 (−1.78 to 4.21)	0.42
Craniofacial skeletal parameters								
SNA, mean (SD), °	79.7 (3.5)	79.2 (3.6)	−0.53 (−1.16 to 0.10)	79.9 (4.3)	79.9 (3.9)	0.03 (−1.49 to 1.54)	−0.60 (−2.02 to 0.82)	0.40
SNB, mean (SD), °	73.3 (3.8)	74.4 (3.5)	1.04 (0.42 to 1.65) **	73.1 (3.7)	73.3 (3.3)	0.17 (−1.03 to 1.36)	0.93 (−0.23 to 2.09)	0.11
ANB, mean (SD), °	6.4 (1.7)	4.8 (1.9)	−1.53 (−2.09 to −0.97) ***	6.8 (2.5)	6.6 (2.3)	−0.12 (−0.86 to 0.63)	−2.94 (−5.45 to −0.43)	0.023 *
MP-SN, mean (SD), °	36.3 (5.4)	37.2 (5.1)	0.92 (−0.04 to 1.89)	38.5 (4.6)	38.5 (3.8)	0.02 (−1.32 to 1.36)	0.42 (−1.05 to 1.89)	0.57
MP-FH, mean (SD), °	28.9 (7.5)	29.0 (7.5)	0.11 (−0.99 to 1.21)	29.7 (4.1)	30.7 (4.9)	1.00 (−0.46 to 2.45)	−0.94 (−2.69 to 0.81)	0.28
Measurements of the upper airway from CBCT								
Total airway volume, mean (SD), mm^3^	11,725.4 (3306.7)	14,422.3 (5027.0)	2696.83 (809.09 to 4584.57) **	9794.7 (2953.1)	13,538.6 (3779.5)	3743.94 (1745.84 to 5742.04) **	−790.11 (−3666.88 to 2086.65)	0.58
Nasopharynx sagittal area, mean (SD), mm^2^	99.2 (31.0)	133.5 (57.5)	34.33 (−5.99 to 74.66)	66.9 (55.6)	154.9 (44.1)	88.00 (55.88 to 120.12) ***	−28.16 (−70.07 to 13.76)	0.18
Nasopharynx volume, mean (SD), mm^3^	2935.7 (700.0)	3953.3 (1262.7)	1017.58 (183.84 to 1851.33) *	1859.6 (1059.9)	3651.1 (1239.2)	1791.56 (1173.72 to 2409.41) ***	−271.37 (−1343.83 to 801.10)	0.61
Palatopharynx sagittal area, mean (SD), mm^2^	230.5 (60.7)	254.1 (62.7)	23.58 (−6.26 to 53.43)	194.5 (60.4)	232.9 (56.1)	38.44 (4.39 to 72.49) *	2.21 (−39.43 to 43.86)	0.9
Palatopharynx volume, mean (SD), mm^3^	3896.7 (1505.7)	4760.7 (1986.4)	864.00 (49.80 to 1678.20) *	3116.3 (1129.7)	4641.5 (1831.4)	1525.25 (604.75 to 2445.76) **	−544.44 (−1838.67 to 749.80)	0.40
Glossopharynx sagittal area, mean (SD), mm^2^	143.8 (33.9)	170.1 (38.6)	26.25 (4.52 to 47.98) *	149.7 (46.4)	134.3 (30.3)	−15.44 (−38.92 to 8.04)	37.99 (13.53 to 62.45)	0.004 **
Glossopharynx volume, mean (SD), mm^3^	2160.5 (877.8)	2618.0 (1366.3)	457.50 (−185.57 to 1100.57)	1913.4 (754.9)	2304.0 (583.6)	390.63 (−131.94 to 913.19)	190.80 (−545.98 to 927.59)	0.60
Laryngopharynx sagittal area, mean (SD), mm^2^	172.4 (81.2)	190.7 (97.8)	18.25 (−15.46 to 51.96)	177.6 (64.5)	193.7 (56.5)	16.13 (−20.46 to 52.71)	0.53 (−46.35 to 47.41)	0.98
Laryngopharynx volume, mean (SD), mm^3^	3111.1 (1423.1)	3509.0 (1675.3)	397.92 (−344.21 to 1140.05)	4338.9 (4339.4)	3348.6 (1216.5)	−990.31 (−3335.26 to 1354.63)	265.82 (−874.47 to 1406.09)	0.64

Abbreviations: CI: Confidence Interval; AHI: apnea–hypopnea index; LSaO_2_: lowest oxygen saturation; CBCT: cone-beam computed tomography. SNA, °: angular indicator for assessment of maxillary protrusion; SNB, °: angular indicator for assessment of mandibular protrusion; ANB, °: angular indicator for assessment of the sagittal relationship between the jaws; MP-FH, °: angle between the mandibular plane and Frankfort plane; MP-SN, °: angle between the mandibular plane and SN plane. Intra-group changes were assessed using paired *t*-test; analysis of covariance (ANCOVA) was used for comparisons between groups. * *p* < 0.05; ** *p* <0.01; *** *p* < 0.001.

## Data Availability

The data presented in this study are available on request from the corresponding author. The raw data cannot be publicly deposited due to patient privacy and ethical restrictions.

## Supplementary Materials

The following supporting information can be downloaded at: https://www.mdpi.com/article/10.3390/dj14080511/s1, Table S1: CONSORT 2025 checklist [41].

## Abbreviations

The following abbreviations are used in this manuscript:

DOT	Dentofacial Orthopedic Treatment
AT	Adenotonsillectomy
OSA	Obstructive Sleep Apnea
AHI	Apnea–Hypopnea Index
RME	Rapid Maxillary Expansion
MA	Mandibular Advancement
CSA	Central Sleep Apnea
BMI	Body Mass Index
PSG	Polysomnography
LSaO_2_	Lowest oxygen saturation
CBCT	Cone-Beam computed tomography
FAS	Full Analysis Set
PPS	Per-Protocol Set
